# Findings in pseudophakic eye that developed liquefied aftercataract-like substance one day after vitrectomy

**DOI:** 10.1016/j.ajoc.2022.101615

**Published:** 2022-06-11

**Authors:** Akiko Ota, Ichiro Ota, Shu Kachi, Goichiro Miyake, Kensaku Miyake, Mineo Kondo

**Affiliations:** a Department of Ophthalmology, Mie University Graduate School of Medicine, Tsu, Japan; b Shohzankai Medical Foundation, Miyake Eye Hospital, Nagoya, Japan

**Keywords:** Capsular block syndrome, Liquefied aftercataract, Vitrectomy, Diabetic retinopathy, Cataract surgery

## Abstract

**Purpose:**

To report our findings in a case that had an accumulation of a translucent fluid between the intraocular lens (IOL) and posterior lens capsule one day after vitrectomy for a vitreous hemorrhage.

**Observations:**

A 67-year-old woman was diagnosed with diabetes 20 years before the vitrectomy and was treated with panretinal photocoagulation (PRP) for proliferative diabetic retinopathy (PDR) 14 years earlier. She underwent cataract surgery with an implantation of an IOL 4 years earlier. She was referred to our hospital because of a vitreous hemorrhage, and we performed uneventful vitrectomy. However, the day after the operation, a translucent liquid substance that resembled liquefied aftercataract was observed in the lens capsule bag. With time, the liquid substance became cloudy. The opacification progressed for two years after the vitrectomy, and her visual acuity decreased. We then performed neodymium: YAG (Nd: YAG) laser posterior capsulotomy, and the cloudy liquid dispersed into the vitreous and the visual acuity improved.

**Conclusions and importance:**

Our findings indicate that liquified aftercataract-like substance can form after vitrectomy in a pseudophakic eye. We suggest that the aqueous humor might flow into the space behind the IOL during or just after the vitrectomy and was trapped behind the IOL optics. Then, the proliferating lens epithelial cells might be dissolved forming the white liquid substance immediately after the surgery.

## 1 Introduction

The presence of liquefied aftercataract substance in the lens capsular bag is a late complication of standard cataract surgery, and it is characterized by the presence of a milky white substance in the closed chamber formed by the optics of an implanted intraocular lens (IOL) and the posterior lens capsule.^1^ Because it takes several years after the cataract surgery for this to occur, liquified aftercataract is categorized as a type of late postoperative capsular block syndrome (CBS).^2^^,^^3^ The presence of a liquefied aftercataract substance rarely results in sight disturbances, but opacities of the posterior lens capsule, e.g., Elschnig's pearls, can reduce the visual acuity.^1^ After neodymium:YAG (Nd:YAG) laser capsulotomy is performed, the milky white substance is dispersed into the vitreous cavity then gradually disappeared. Vitreous opacities after YAG laser capsulotomy remain in some cases but seldom require vitrectomy.^1^

We report our findings in a case in which liquefied aftercataract substance was detected on the day after a vitrectomy in a diabetic patient who had undergone IOL implants 4 years earlier.

## 2 Case report

A 67 years-old woman with a 20-year history of diabetes underwent panretinal photocoagulation (PRP) for proliferative diabetic retinopathy (PDR) in both eyes in 2005. She then had cataract surgery with intraocular lens implantation on both eyes in 2015. Four years later in 2019, she complained of decreased vision of the right eye and was diagnosed with a vitreous hemorrhage. She was then referred to our hospital for surgical treatment.

**Fig. 1:**
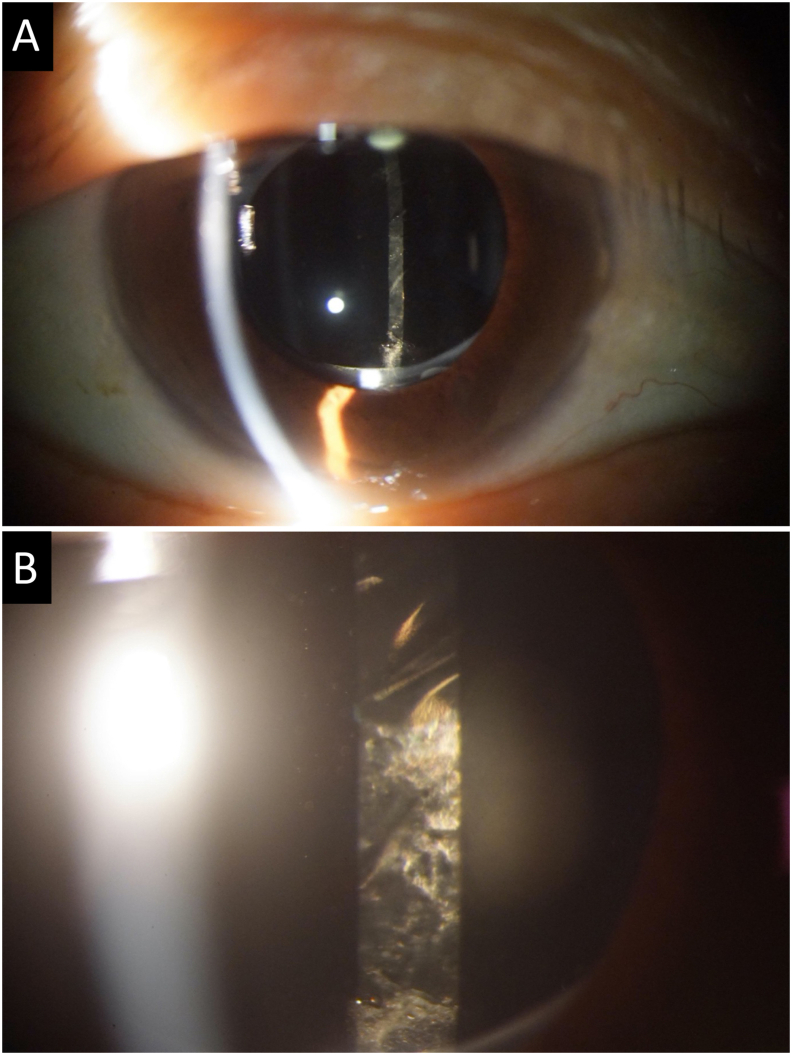
**Slit-lamp images of an eye taken at the first examination one day after the surgery**, **A:** Slit-lamp image shows that the intraocular lens (IOL) is completely implanted in the posterior capsular bag. **B:** Slit-lamp image of the posterior surface of the lens before the vitrectomy. Opacities can be seen coating the posterior lens capsule.

At the initial examination, her best-corrected visual acuity (BCVA) was 20/50 (+sph 1.25 -cyl 2.50 Ax 105) in the right eye (OD) and 20/20 (+sph 1.25 -cyl 2.50 Ax 90) in the left eye (OS). The intraocular pressure (IOP) was 14 mmHg OD and 15 mmHg OS. Slit-lamp examination of both eyes showed that the anterior segments were clear, and the IOL was centered and fixed in the capsular bag (Fig. 1A). The posterior capsular surface appeared adherent to the posterior surface of the optic of the IOL, and no aftercataract liquid was present (Fig. 1B). Funduscopic examination of her right eye revealed a moderate vitreous hemorrhage and tractional retinal membranes accompanied by mild neovascularization on the nasal side of the optic disc. Laser scars from the PRP were also observed.

**Fig. 2:**
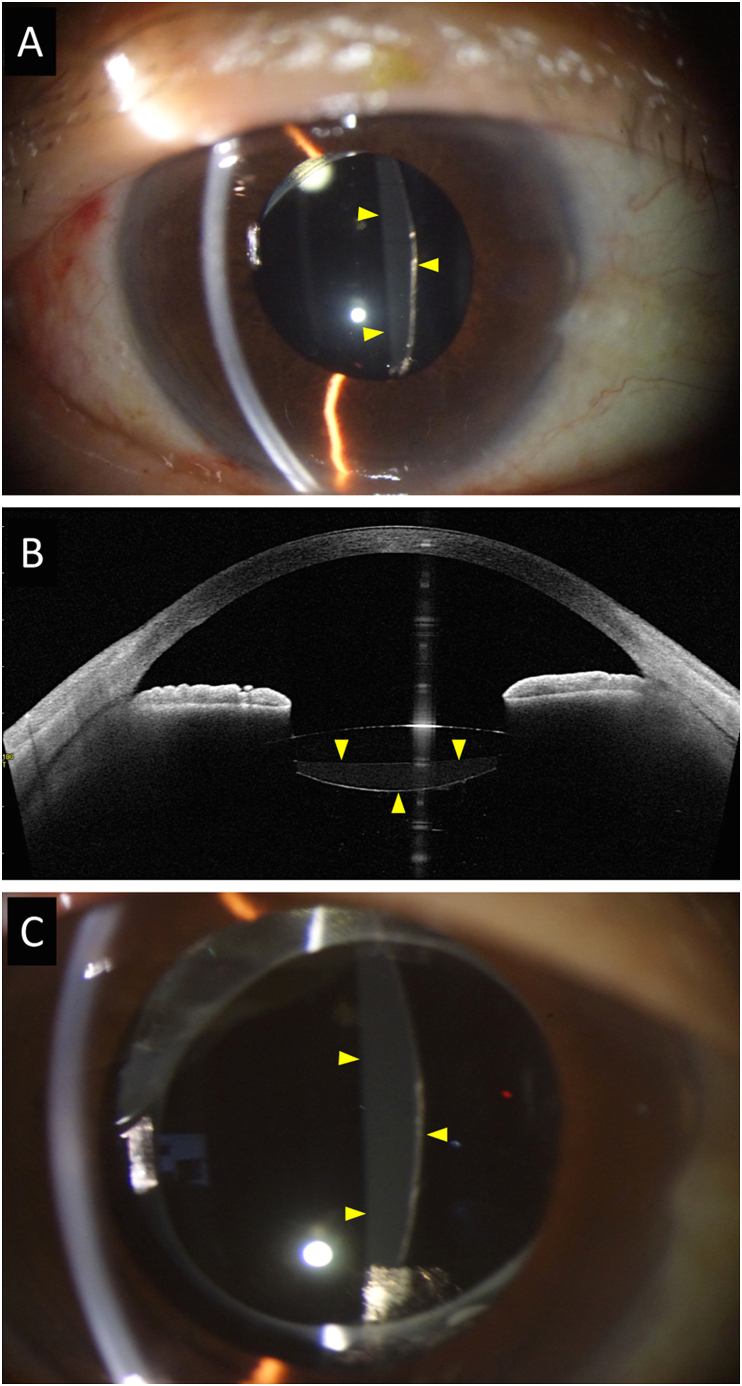
**Slit-lamp images and anterior segment-optical coherence tomographic (AS-OCT) image after pars plana vitrectomy**. **A:** Slit-lamp image shows a distention of posterior lens capsule on the first day after the vitrectomy. Photograph shows mild fibrosis on the edges where continuous curvilinear capsulorhexis had been performed and the presence of Elschnig's pearls on the inferior part of the optic of the IOL. Yellow arrowheads show the area of liquified aftercataract-like substance. **B:** Anterior segment-optical coherence tomographic (AS-OCT) image one day after pars plana vitrectomy. A liquid substance can be seen between the posterior surface of the optic of the IOL and the posterior lens capsule (yellow arrowheads). This image resembles that of liquefied aftercataract although the density appears to be lower than the typical liquefied aftercataract. **C:** Slit-lamp photograph taken 5 months after the vitrectomy. The distention of the posterior lens capsular bag is unchanged (yellow arrowheads). (For interpretation of the references to colour in this figure legend, the reader is referred to the Web version of this article.)

We performed pars plana vitrectomy (PPV) without posterior capsulotomy, and the entire procedure was uneventful. Slit-lamp examination on the first day after surgery showed that the posterior lens capsular bag behind the optic of the IOL was distended and protruded smoothly into the vitreous cavity. This distention seemed to be thickest in the center of the IOL and thinner toward the periphery (Fig. 2A). Although this appearance was similar to that of cases of liquefied aftercataract substance, the material appeared to be less dense than the ones we had experienced earlier. The presence of this substance was confirmed by anterior segment optical coherence tomography (AS-OCT; Fig. 2B). Postoperative ophthalmoscopy showed that the vitreous hemorrhage was absent and the contractile membranes had been peeled off of the retina.

**Fig. 3:**
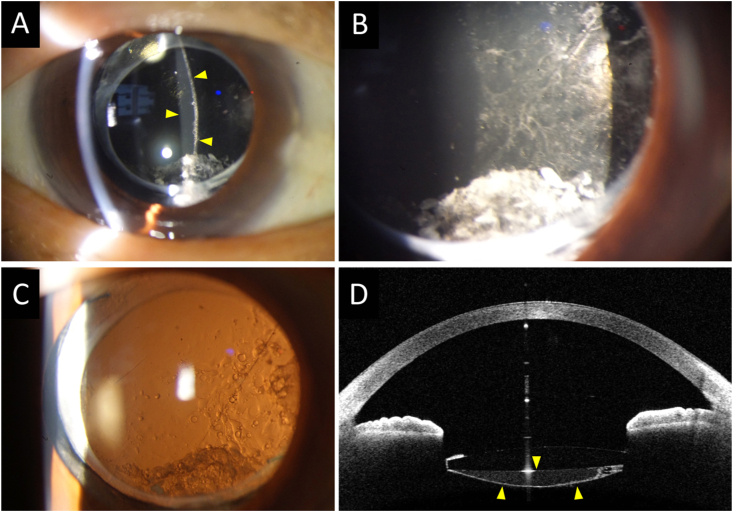
**Slit-lamp photographs and AS-OCT image 2.5 years after the vitrectomy**. **A:** Slip-lamp photograph taken approximately 2.5 years after the vitrectomy showing that the distention of the posterior lens capsular bag does not appear to have changed. Yellow arrowheads show the area of liquified aftercataract-like substance. **B:** Slit-lamp photograph show that the liquid accumulation behind the IOL appears cloudy which resembles more closely the typical liquefied aftercataract than before. **C:** Fibrosis along the edge of CCC and Elschnig's pearls have become more conspicuous. **D:** AS-OCT image showing the liquid substance behind the IOL having a higher density (yellow arrowheads). (For interpretation of the references to colour in this figure legend, the reader is referred to the Web version of this article.)

**Fig. 4:**
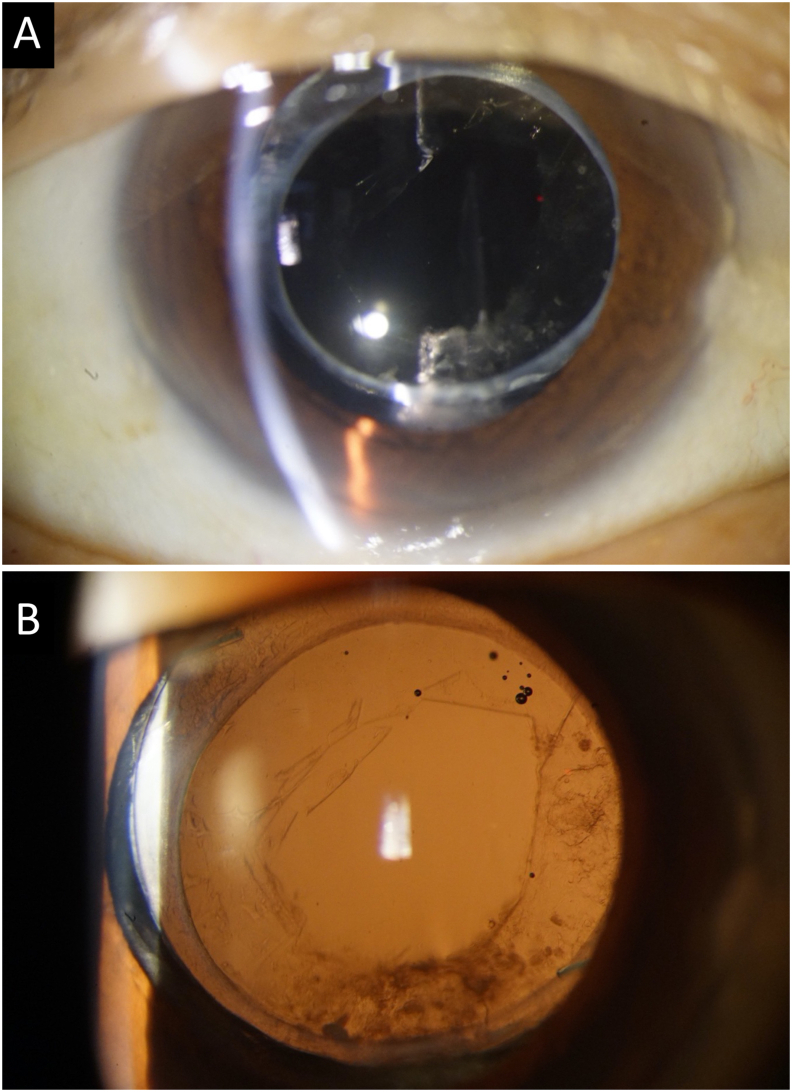
**Slit-lamp photograph after Nd:YAG posterior capsulotomy.** **A & B:** After round pattern Nd:YAG laser posterior capsulotomy, the liquid substance has disappeared.

The distention of the capsular bag remained unchanged 5 months postoperatively (Fig. 2C), and the BCVA was 20/20 (+sph 0.50 -cyl 1.00 Ax 90). Two years later, her BCVA decreased to 20/32 (+sph 0.75 -cyl 2.00 Ax 100). At this examination, she was found to have a posterior capsule opacity and the Elschnig's pearl was larger than two years earlier (Fig. 3A–D). The liquid substance behind the IOL was cloudy, and it appeared almost identical to the liquified aftercataract substance detected after cataract surgery with IOL implantation (Fig. 3A–D). Because the reduction of vision was believed to be due to the posterior capsule opacity, we performed Nd:YAG laser capsulotomy on the same day. The liquidsubstance quickly dispersed into vitreous cavity, and the whole procedure was uneventful (Fig. 4A & B). The patient's BCVA improved to 20/16 (+sph 1.5 -cyl 2.00 Ax 95) after the capsulotomy.

At her last examination, her BCVA was unchanged at 20/16 (OD), and the ophthalmoscopic appearance had also not changed.

## 3 Discussion

CBS is a complication of cataract surgery in which the fluid accumulates in the capsular bag between the IOL and the posterior capsule. There are three types of CBS: intraoperative, early postoperative, and late postoperative.^2^^,^^3^ In these cases, the opening from the posterior chamber into the anterior chamber is blocked by the nucleus of the lens or the optic of the IOL which causes a liquid substance to accumulate in the capsule bag.

Typically, early postoperative CBS occurs on either the day after or within several weeks after the cataract surgery. In these cases, the anterior opening of the capsular bag created by the CCC is blocked by the optic of the IOL. This results in the accumulation of transparent fluid in the bag. The cause of the CBS is believed to be due to the trapping of viscoelastic substance in the capsular bag after the surgery or due to the presence of residual lens epithelial cells and lens cortex trapped in the capsular bag. These induce an increase in the oncotic or colloidal osmotic pressure which draws more fluid across the capsular membrane into the capsular bag.^4^, ^5^, ^6^

The usual liquefied aftercataract complication is categorized as a late postoperative CBS because it takes, on an average, of 3.8 years to occur.^1^^,^^2^ Slit-lamp biomicroscopy of these cases shows a homogeneous milky white substance within a closed chamber formed by the fibrosis along the entire circumference of the opening between the anterior surface of the IOL and edge of the capsulorhexis opening.^2^^,^^3^ These findings suggest that the posterior surface of the IOL and posterior capsule are in contact but not completely adherent even after several years. It can also be explained by the fact that they can be separated during IOL extraction or exchange procedures. We assume that the liquid substance accumulated in this space in our case as well. The ophthalmoscopic appearance of our case resembled a late postoperative CBS more than an early postoperative CBS. An acute development of liquefied aftercataract seen in our patient does not appear to be due to infection because panuveitis was not induced by the YAG laser capsulotomy.

The milky white substance of the typical liquefied aftercataract has been shown to consist of lens proteins related to proliferating lens epithelial cells, extracellular matrix, and liquified cortical matter.^7^, ^8^, ^9^ Nevertheless, it is difficult to believe that this substance was developed in just one day after the vitrectomy in our case. We suggest that the aqueous humor might flow into the space behind the IOL through a small anterior lens capsule opening during or just after the vitrectomy. Then, the liquid substance becomes trapped behind the IOL presumably because the optic of IOL can have act like a valve within the CCC opening. Later, the proliferating lens epithelial cells such as Elschnig's pearls may have been dissolved by the aqueous humor forming the white liquid substance immediately after the surgery. In fact, our case had mild Elschnig's pearls on the inferior part of the posterior lens capsule before the vitrectomy (Fig. 1B).

The findings in our case differed from that of the typical liquefied aftercataract cases by the fact that the liquid substance was less dense initially. However, it gradually changed over a two-year period and appeared as typical liquefied aftercataract substance. Atypical liquified aftercataract has also been reported by Namiki et al. as localized liquefied aftercataract, in which milky white substance accumulated not evenly in the entire capsular bag but in localized closed chamber between the optic of the IOL and the posterior lens capsule.^10^

We suggest that our case can be categorized into a new subtype of early postoperative CBS in a pseudophakic eye. Furthermore, the components of the substance may be different from the typical liquefied aftercataract even though they look alike.

There is one major limitation in this case. We reported our findings in only one case. However, we believed that this information will help other clinicians to be aware of similar occurrences. Collectively, the information should be helpful in determining the mechanism for the development of the fluid in the capsular bag soon after vitrectomy.

In conclusion, we reported a case that had a liquified aftercataract-like substance between the IOL and posterior lens capsule one day after vitrectomy. We suggest that the aqueous humor might have intruded into the space behind the IOL during or just after the vitrectomy and was trapped behind the IOL optics. Then the proliferating lens epithelial cells might be dissolved forming the white liquid substance immediately after the surgery.

## Patient consent

Written informed consent was obtained from the patient for publication of this case report and any accompanying images. A copy of the written consent is available for review by the Editor of this journal.

## Funding

No funding or grant support.

## Author contributions

AO, KM and MK wrote the main manuscript. AO, IO, SK, GM collected the data. All authors reviewed the manuscript.

## Proprietary interest

None for all authors.

## Declaration of competing interest

None for all authors.
